# Hashimoto thyroiditis is more frequent than expected when diagnosed by cytology which uncovers a pre-clinical state

**DOI:** 10.1186/1756-6614-3-11

**Published:** 2010-12-20

**Authors:** Anca Staii, Sarah Mirocha, Kristina Todorova-Koteva, Simone Glinberg, Juan C Jaume

**Affiliations:** 1 Endocrinology, Diabetes and Metabolism, Department of Medicine, School of Medicine and Veterans Affairs Medical Center, University of Wisconsin-Madison, Madison WI 53792, USA

## Abstract

**Background:**

Our Thyroid-Multidisciplinary Clinic is a large referral site for thyroid diseases. Thyroid biopsies are mainly performed for thyroid cancer screening. Yet, Hashimoto thyroiditis (HT) is being too frequently diagnosed. The prevalence of HT is reported as 0.3-1.2% or twice the prevalence of type 1 diabetes. However, the prevalence of HT confirmed by cytology is still uncertain. To evaluate different aspects of thyroid physiopathology including prevalence of Hashimoto's, a database of clinical features, ultrasound images and cytology results of patients referred for FNA of thyroid nodules was prospectively developed.

**Methods:**

We retrospectively studied 811 consecutive patients for whom ultrasound guided thyroid FNA biopsies were performed at our clinic over 2.5 year period (Mar/2006-Sep/2008).

**Results:**

The analysis of our database revealed that from 761 patients, 102 (13.4%) had HT, from whom 56 (7.4%) were euthyroid or had sub-clinical (non-hypothyroid) disease, and 46 (6%) were clinically hypothyroid.

**Conclusions:**

This is the first study to show such a high prevalence of HT diagnosed by ultrasound-guided FNA. More strikingly, the prevalence of euthyroid HT, appears to be >5% similar to that of type 2 diabetes. Based on our results, there might be a need to follow up on cytological Hashimoto's to monitor for thyroid failure, especially in high risk states, like pregnancy. The potential risk for thyroid cancer in patients with biopsy-proven inflammation of thyroid epithelium remains to be established prospectively. However, it may explain the increased risk for thyroid cancer observed in patients with elevated but within normal TSH.

## Background

Hashimoto thyroiditis is the most common cause of hypothyroidism in the United States [1]. With a 5-10 time preference over men, the reported prevalence in white women is in the 1-2% range [2].

Etiology and pathogenesis of Hashimoto thyroiditis are still elusive. Moreover, little is known about progression of euthyroid to hypothyroid Hashimoto's. At least in children, disease progression from euthyroid to hypothyroid Hashimoto thyroiditis has been suggested [3]. Also, available evidence relating the progression of sub-clinical to overt hypothyroidism in adults has been rated as good [4]. Hence, it is conceivable that a euthyroid stage of Hashimoto thyroiditis exists and that progression to a full-blown disease stage is a matter of time.

Since there is growing evidence that unrecognized hypothyroidism is deleterious, early diagnosis of Hashimoto thyroiditis would be advantageous in predicting thyroid failure. Specifically, it is well known that maternal thyroid status assessment and treatment improves fetal outcomes and neuropsychological developmental of the newborn [5].

The University of Wisconsin Thyroid Multidisciplinary Clinic is a large referral site for thyroid diseases in the Midwest. A continuously increasing number of thyroid biopsies are being performed every year for cancer screening. Yet, Hashimoto thyroiditis is being too frequently diagnosed. The prevalence of Hashimoto thyroiditis is reported to be approximately twice that of type 1 diabetes. However, the prevalence of Hashimoto thyroiditis confirmed by cytology has never been documented in a large cohort of patients with ultrasound detectable nodules. To evaluate different aspects of thyroid physiopathology, a database of clinical features, ultrasound images and cytology results of patients referred for fine needle aspiration of thyroid nodules was prospectively developed. In this paper we probed our database for the frequency and characteristic of patients diagnosed with Hashimoto thyroiditis while being referred for thyroid cancer screening.

## Methods

### Thyroid Database

**Figure 1:**
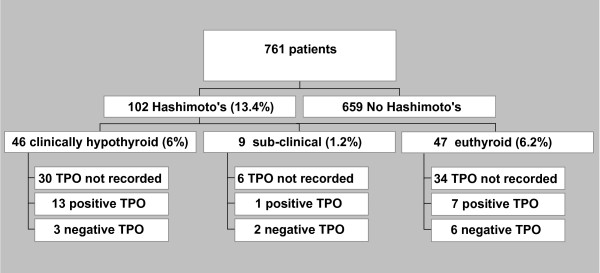
**Patients' allocation diagram**.

From March 2006 until September 2008, 811 patients underwent ultrasound guided fine needle aspiration (FNA) biopsy of thyroid nodules for screening of thyroid cancer. After excluding 50 patients who were either no-show or their specimens were non-diagnostic, we retrospectively studied 761 consecutive patients for which ultrasound guided thyroid FNA biopsies were performed at our clinic (Figure 1). All FNA samples were reviewed by cytopathologists at our institution.

The Hashimoto thyroiditis cohort consisted of 102 (13.4%) patients (659 out of 761 did not have cytological Hashimoto's diagnosis) for which 46 (6%) were identified as having clinical disease (i.e. diagnosed hypothyroid on thyroid hormone replacement and with cytological Hashimoto's diagnosis), 9 (1.2%) as having sub-clinical hypothyroidism (as defined by normal thyroid hormones with above normal [usually less than 10 ng/dl] TSH and with cytological Hashimoto's diagnosis) and 47 (6.2%) as having euthyroid autoimmunity (as defined by normal thyroid hormones with normal [0.45-4.12 ng/dl] TSH but with cytological Hashimoto's diagnosis). For all patients, data were collected for: age, gender, nodule size and number of nodules, levels of TSH/FT4, presence of TPO (thyroid peroxidase) autoantibodies, family history of Hashimoto's, goiter, and autoimmune diseases. The collection of patient's data and subsequent analysis was approved by University of Wisconsin Human Subjects Institutional Review Board.

### Statistical Analysis

Data analysis was conducted using Excel X2 and Fisher's exact test and Student t-test were used when appropriate. Statistical significance was defined as P < 0.05.

## Results

### Patients Characteristics

**Figure 2:**
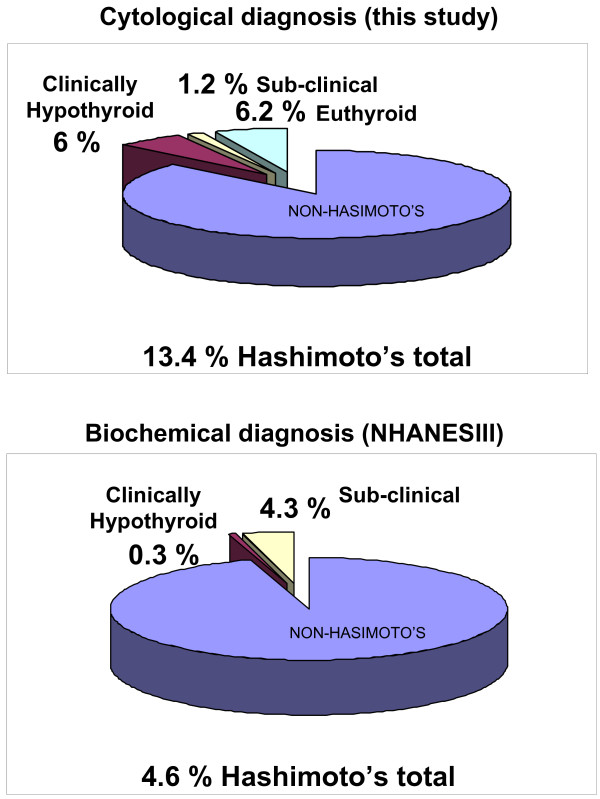
**Prevalence comparison**. Chart comparison is provided between this study and NHANES III (9). The total population of NHANES III study is used as a reference for the comparison of clinical and subclinical disease in both studies.

Patients were referred to the University of Wisconsin Thyroid Multidisciplinary Clinic usually for evaluation of an already diagnosed thyroid problem. Thyroid nodules ≥ 1 cm were biopsied under ultrasound guidance mainly to screen for thyroid cancer. On-site cytopathology was available for every single case. Endocrine surgery referral was also available as needed during the same encounter. A database was started with the inauguration of the clinic and recruitment is ongoing. Of the first 811 patients' data analyzed, an unexpected large number of patients with positive Hashimoto thyroiditis cytology were identified. The analysis of our database revealed that from 761 patients, 102 (13.4%) had cytology proven Hashimoto thyroiditis. Patients' characteristics of this cohort are shown on table 1. Hashimoto thyroiditis cohort consisted of 94 female and 8 male with mean (± SD) age of 47 ± 14 years. Out of these 102 patients with cytology-proven Hashimoto thyroiditis, 47 (6.2%) were euthyroid, 9 (1.2%) had sub-clinical hypothyroidism, and 46 (6%) had clinical hypothyroidism and were on thyroid hormone replacement (Table 1 and Figure 2). The number of nodules was 1.43 ± 0.54 average for the clinically hypothyroid subgroup, 1.77 ± 0.66 average for the sub-clinical subgroup (p < 0.05 when compared to clinically hypothyroid subgroup) and 1.58 ± 0.71 for the euthyroid subgroup. The size of the nodules was 1.42 ± 0.42; and 1.55 ± 0.81; for the clinical and sub-clinical subgroups respectively and 1.69 ± 0.74 (p < 0.05, also Table 1) for the euthyroid subgroup. Although differences between nodules sizes of clinically hypothyroid versus sub-clinical subgroups appear significant, the result of this comparison is skewed by the low number of patients in the sub-clinical subgroup as well as the wider range. Larger size of euthyroid subgroup nodules was however statistically significant when compared to clinically hypothyroid subgroup nodules both subgroups with similar number of nodules. Another significance difference was also observed in the comparison of TSH values between euthyroid and clinically hypothyroid subgroups as expected. The clinically hypothyroid subgroup had an average TSH of 8 ± 2.61 ng/dl while the euthyroid subgroup had a lower TSH of 2.18 ± 1.14 ng/dl. The sub-clinical subgroup had average TSH value of 6.73 ± 1.26 ng/dl. All nodules were reported as benign. Of note, although patients with clinical hypothyroidism were all taking thyroid hormone, none in the sub-clinical or the euthyroid subgroups were on thyroid hormone replacement. FT4 (free T4) in the clinically hypothyroid subgroup was found to be 1 ± 0.36; 0.96 ± 0.11 in the sub-clinical and 0.92 ± 0.11 in the euthyroid subgroup. FT4 however was not recorded in 52% of the clinically hypothyroid subgroup and in 56/57% of the sub-clinical/euthyroid subgroups respectively. Not recorded also, was the presence or absence of TPO autoantibodies in 30 of 46 patients with clinical hypothyroidism, 6 of 9 patients with sub-clinical and 34 of 47 with euthyroid Hashimoto's. However out of the recorded TPO autoantibodies, 13 of 16 (81.3%) were positive in the clinically hypothyroid subgroup while 8 of 16 (50%) were positive in the sub-clinical/euthyroid subgroups (also in Table 1). There were no significant differences among euthyroid, sub-clinical and clinically hypothyroid Hashimoto thyroiditis subgroups in terms of age, gender, family history of Hashimoto's, goiter, and/or autoimmune diseases (also in Table 1).

**Table 1:** Patients Characteristics - Hashimoto Thyroiditis Patients*

	Clinically hypothyroid	Sub-clinically hypothyroid	1^st ^P-value**	Euthyroid	2^nd ^P-value**
FNA patients	46	9		47	
Age (years)	47 ± 12	50 ± 17	0.53	46 ± 15	0.72
Gender					
Male	2 (4%)	2 (22%)	0.15	4 (9%)	0.68
Female	44 (96%)	7 (78%)	0.79	43 (92%)	1
Nodule					
# of nodules	1.43 ± 0.54	1.77 ± 0.66	*<0.05*	1.58 ± 0.71	0.26
Size	1.42 ± 0.42	1.55 ± 0.81	0.48	1.69 ± 0.74	*<0.05*
TSH(0.45-4.12 ng/dl)	8 ± 2.61	6.73 ± 1.26	0.16	2.18 ± 1.14	*<0.05*
FT4	1 ± 0.36	0.96 ± 0.11	0.74	0.92 ± 0.11	0.15
Not Recorded	24 (52%)	5 (56%)		27 (57%)	
Recorded	22 (48%)	4 (44%)		20 (43%)	
TPO antibodies					
Not Recorded	30	6		34	
Positive	13	1	0.62	7	0.56
Negative	3	2	0.27	6	0.45
FHx of Hashimoto's/goiter/autoimmunity	18 (39%)	4 (44%)	1	10 (21%)	0.19

* Values reported as mean ± SD.

** Fisher exact test and Student t-test. 1^st ^P-value is between Clinically and Sub-clinically hypothyroid. 2^nd ^P-value is between Clinically hypothyroid and Euthyroid. Statistically significant values are shown in *italics*.

### Hashimoto thyroiditis more common than expected

**Figure 3:**
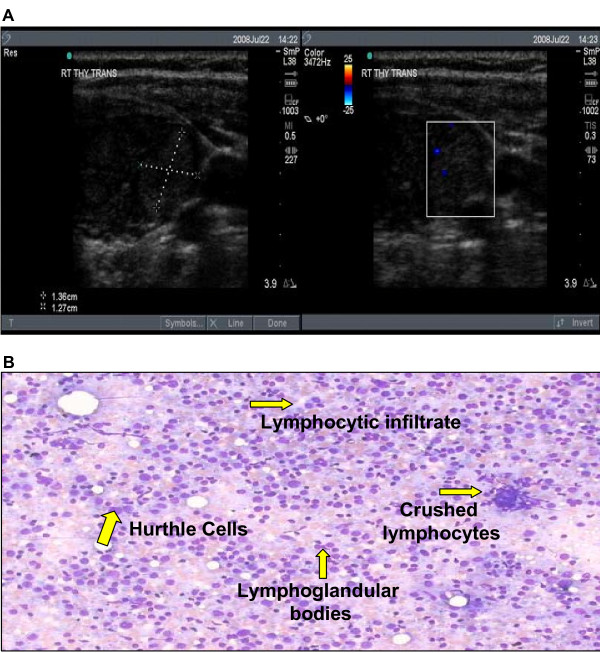
**A. Ultrasound image of euthyroid Hashimoto thyroiditis**. A representative ultrasound is shown of an asymptomatic, euthyroid patient's thyroid nodule cytologically diagnosed Hashimoto thyroiditis. Note the minimal color flow Doppler identified within the nodule. **B. Cytopathology. **Cytological characteristics of a thyroid nodule with typical diagnostic features of Hashimoto's thyroiditis (i.e. lymphocytic infiltrate, Hurthle cells, lymphoglandular bodies and crushed lymphocytes).

Although the prevalence of Hashimoto thyroiditis diagnosed by ultrasound guided FNA cytology has not been established, the prevalence found in our cohort was unexpectedly high in comparison with that reported for Hashimoto's hypothyroidism and subclinical disease combined (Figure 2). The reasons for these differences in prevalence estimates are not obvious. Hence we considered potential factors that might have biased the results. Among them, the clinical indications for the biopsies and the cytological criteria for Hashimoto thyroiditis diagnosis were reviewed for all cases. Clinical indications for biopsies of nodules were based on the American Thyroid Association guidelines for size criteria (see nodule sizes in Table 1) which were determined by ultrasound before and confirmed during the biopsy (Figure 3A as example). Standard cytological characteristics for diagnosis were also followed by our cytologists as described by Kini [6]. The cytological diagnosis of Hashimoto's thyroiditis relied on the presence of both an inflammatory infiltrate accompanied by thyroid follicular cells. Mixed population of lymphocytes, sometimes represented in the form of crushed lymphocytes and lymphoglandular bodies (cytoplasmic debris) together with the presence of Hurthle cells were described in all cytology reports (Figure 3B as example). All of these characteristics were considered in the diagnosis of each and every patient in our cohort. Lastly, our referral population is broad but representative of an area considered iodine sufficient.

## Discussion

This is the first study to show such a high prevalence of Hashimoto thyroiditis diagnosed by ultrasound-guided FNA cytology on a somewhat large cohort of patients. Based on our study, the prevalence of cytology-proven Hashimoto thyroiditis appears to be >10% in patients with thyroid nodules. Given that the prevalence of thyroid nodules on ultrasonography or autopsy data is as high as 50% [7], such a high prevalence of Hashimoto thyroiditis diagnosed by cytology is noteworthy. More strikingly, the prevalence of euthyroid, non-previously diagnosed, cytology-proven Hashimoto thyroiditis (euthyroid autoimmunity), appears to be >5% in our study (Figure 1 and 2). This condition has not been previously defined. For comparison, its prevalence is similar to that of type 2 diabetes which is considered to be a health care crisis.

Weetman [2] reported clinical Hashimoto thyroiditis prevalence rate at 1 in 182 or 0.55% in the US. In the UK, Tunbridge et al [8] reported an overall Hashimoto thyroiditis prevalence of 0.8%. However, based on our study, the cytology of Hashimoto thyroiditis seems to be much more prevalent, at 13.4%. This difference may be partially explained by the fact that for diagnosing clinical Hashimoto thyroiditis, abnormally elevated TSH, low thyroid hormones [2,8] and the confirmatory presence of thyroid autoantibodies are usually accounted for. We hypothesized then, that cytological diagnosis of Hashimoto's may precede clinical diagnosis. Interestingly however, in most organ specific autoimmune diseases, humoral immunity heralds tissue infiltrative damage. Hence, we expected the diagnosis of Hashimoto's by cytology to be less but not more common than the antibody diagnosis especially on early stages of the disease (Figure 2).

In an attempt at differentiating better the apparent stages of Hashimoto thyroiditis, we divided our cytology-proven Hashimoto's cohort into three subgroups: clinically hypothyroid, sub-clinical and euthyroid. For these distinctions, we used the clinical definition of hypothyroid Hashimoto thyroiditis as that occurring in patients usually with TSH greater than 10 ng/dl at diagnosis, with low free thyroid hormones and on thyroid hormone replacement. Based on NHANES III study [9] normative data for TSH distribution (reference population of 13,344, 95% of TSH between 0.45-4.12 ng/dl), abnormally high TSH (but usually less than 10 ng/dl) with normal thyroid hormones was used to define sub-clinical hypothyroidism. Patients with those characteristics were assigned to the sub-clinical subgroup in our study. A third subgroup classified as euthyroid included patients with normal thyroid hormones and normal TSH.

Aside from the unexpected observation of the high prevalence of Hashimoto thyroiditis by cytology, especially in euthyroid patients, the lack of cytological correlation with TPO autoantibody positivity is noteworthy. As mentioned before, the hallmark in the diagnosis of Hashimoto thyroiditis is the presence of TPO autoantibodies [10]. Yet, only about half of the patients tested for anti-TPO in the euthyroid subgroup were positive. On the other hand and although non-statistically significant because of the small number reported, the clinically hypothyroid subgroup had most (13 out of 16 tested) patients positive for anti-TPO. Similarly, Poropatich et al., [11] found that anti-TPO and/or antithyroglobulin antibody titers were present in only 50% of the patients with euthyroid, cytology-proven Hashimoto thyroiditis, a finding never reproduced by these or other authors in the literature.

Given the wide range of normal values for TSH (1 fold) and the variability on the presence of TPO autoantibodies, it is conceivable that early Hashimoto's autoimmune process might be clinically missed. These issues, together with the awareness that sub-clinical and clinical hypothyroidism associates with cardiovascular and neuropsychiatric morbidities, make finding high prevalence of Hashimoto thyroiditis on cytology, especially in euthyroid patients clinically significant [12-14].

An important aspect of our findings is the fact that most of the patients in our cohort are pre-menopausal females. Past and more recent studies found that the risk of poor obstetrical outcome is increased with relative thyroxine deficiency [15-18]. Benhadi et al. recently showed that the risk of miscarriage, fetal and neonatal death is increased with higher TSH levels. Moreover, the risk of fetal loss occurred even when maternal free thyroxine levels were normal [16]. Based on our results of high prevalence of cytology-proven Hashimoto thyroiditis and especially high prevalence of euthyroid disease in pre-menopausal women, there might be need of at least follow up of thyroid function on cytologically-proven Hashimoto's in pre-conception through delivery stages.

Positive Hashimoto's cytology clearly represents a state in which inflammation of thyroid epithelium may lead to tissue remodeling. Hence the possibility of acquiring mutations while cells are dividing might be greater. Some studies have suggested that inflammation of thyrocytes could be linked to thyroid cancer [19-21]. For example, Larson et al. showed that phosphatidylinositol 3-kinase phosphorilates Akt, which in turn suppresses pro-apoptotic signals and promotes tumorigenesis, was increased in both Hashimoto thyroiditis and well differentiated thyroid cancer [19]. Furthermore, Borrello et al., showed that RET/PTC oncogene, when exogenously expressed in normal thyrocytes, induces expression of a large set of genes involved in inflammation and tumor invasion (cytokines, matrix-degrading enzymes and adhesion molecules) [20]. There seem to be however, controversial opinions regarding the association of Hashimoto thyroiditis and thyroid cancer. Some studies find Hashimoto thyroiditis as associated with thyroid cancer [22,23] as examples, while others do not report an association [24,25] as examples. Since higher TSH level in patients with thyroid nodules has been found to be associated with risk of differentiated thyroid cancer [26,27], we hypothesized that active remodeling of thyroid epithelium in cytological Hashimoto's may explain in part the increased risk for differentiated thyroid cancer observed in patients with elevated but within normal TSH [26,27].

## Conclusions

We report here that the pathogenesis of Hashimoto thyroiditis is present in an unexpectedly large number of individuals. Moreover, we found that many of these individuals carry a non-clinically manifested process. Biopsy proven Hashimoto thyroiditis in asymptomatic, clinically disease-free patients is a condition still undefined. There are no guidelines as to how to follow these patients. What is their risk of progressing to full-blown Hashimoto's? How would pregnancy affect their thyroid function? Are they at further risk of developing thyroid cancer since their TSH usually runs in the normal but higher quartile? Recognition of this entity emerges as necessary at least in patients at risk for hypothyroid complications (i.e. pregnancy). If the cytology of Hashimoto thyroiditis represents a chronic active inflammatory state, TSH elevations within normal limits may represent a manifestation of a common underlying process.

## List of abbreviations used

HT: Hashimoto thyroiditis; FHx: Family history; FT4: Free T4; FNA: Fine needle aspiration; TPO: Thyroid peroxidase.

## Competing interests

The authors declare that they have no competing interests.

## Authors' contributions

All authors participated in the design of the study. AS and JCJ performed the statistical analysis. JCJ conceived of the study. All authors read and approved the final manuscript.

## Aknowledgements

We would like to thank Drs. Haymart, Heras-Herzig, Gogineni, Bradley, and Poehls for their active participation in the ongoing University of Wisconsin Thyroid Database Study and Dr. Ernest L. Mazzaferri for critical review of this paper. This work was funded in part by NIH and VA grants (JCJ) and presented in part at the 2009 Endocrine Society Annual Meeting, USA.
